# Telithromycin-resistant *Streptococcus pneumoniae*

**DOI:** 10.3201/eid1109.050415

**Published:** 2005-09

**Authors:** Fred W. Goldstein, Barbara Vidal, Marie D. Kitzis

**Affiliations:** * Hospital St. Joseph, Paris, France

**Keywords:** telithromycin resistance, S. pneumoniae, clinical failure, letter

**To the Editor:** In recent years, antimicrobial drug resistance in *Streptococcus pneumoniae* has increased worldwide and is a major health concern. Resistance to β-lactams and macrolides, considered to be first-line therapeutic agents, is particularly high in France and many Asian countries (*1**–**3*). Resistance to new fluoroquinolones is reported with increasing frequency, which emphasizes the need for new effective drugs. Telithromycin, the first member of a new macrolide family, the ketolides, has been developed to overcome macrolide resistance. In vitro data have shown that telithromycin remains active against 98% to 100% of erythromycin-resistant strains (*2**,**3*).

However, resistant mutants have been isolated in vitro, and a few poorly documented clinical failures have been reported in the treatment of pneumococcal infections. We report the first isolation of telithromycin-resistant *S. pneumoniae* from a blood culture after therapy.

An 87-year-old woman was admitted on March 28, 2004, to St Joseph Hospital in Paris with typical upper left lobar pneumonia, as inferred from auscultatory results, radiologic findings, and laboratory data: leukocytes 37,300 cells/μL, C-reactive protein 455 mg/L, and positive urinary pneumococcal antigen (BinaxNOW, Binax, Inc., Portland, ME, USA). She was not febrile. She had been followed for many years for chronic obstructive pulmonary disease (COPD), with acute exacerbation only in 2001. At that time, she was treated with the macrolide roxithromycin, without bacteriologic documentation, in addition to acetylcysteine (3 × 200 mg/d) and aerosolized terbutaline. On March 13, her COPD was exacerbated. On March 20, she visited her general practitioner and received 800 mg/day telithromycin for 5 days without improvement. Because of a cutaneous rash attributed to telithromycin, she received 20 mg prednisolone. After 48 hours, she was admitted to St Joseph Hospital because her respiratory syndrome was aggravated. A blood culture drawn on admission yielded a *S. pneumoniae* serotype 14 with decreased β-lactam susceptibility (MICs: penicillin G: 1 μg/mL; amoxicillin: 0.75 μg/mL; cefotaxime: 0.5 μg/mL, as determined by Etest). The strain was resistant to tetracyclines, cotrimoxazole, macrolides, and lincosamides (erythromycin and clindamycin MIC >32 μg/mL). The MIC of telithromycin, performed on Mueller-Hinton agar + 5% horse blood by serial 2-fold dilution, was equal to 2 μg/mL in air and 8 μg/mL under CO_2_ (0.01–0.03 μg/mL for control strains ATCC 49619 and 10 clinical isolates, including 5 that were MLSB [macrolide-lincosamide-streptogramin B]–resistant). The patient was treated with 100 mg/kg/day intravenous amoxicillin and improved within 48 hours. She was discharged from the hospital 1 week later in good condition but remained a healthy carrier of resistant *S. pneumoniae*.

Resistance to macrolides has been documented in France since our first report in 1978 (*4*). In the last 10 years, resistance has increased to ≈50% of the strains in adults and >70% in children, the highest in the Western world. More than 98% of the strains are of the MLSB phenotype, conferring high-level resistance to macrolides, lincosamides, and streptogramin B, in contrast to the situation in the United States, where most strains are of the *mefE* type (efflux), which confers low-level resistance to 14- and 15-membered macrolides only. However, <2% of the macrolide-resistant strains have a decreased susceptibility to telithromycin (2,3). Resistance to β-lactams is also very frequent (≈50%), particularly in erythromycin-resistant strains (<90%); these figures explain why macrolides may more likely select a penicillin-resistant strain than most β-lactams (*5*). Since resistance to telithromycin was documented before ketolides were introduced in clinical practice, we cannot exclude the possibility that the telithromycin­-resistant strain was selected in 2001, while our patient was treated with roxithromycin.

The clinical impact of macrolide resistance has been occasionally questioned since these antimicrobial agents achieve high tissue and intracellular levels. However, *S. pneumoniae* is an extracellular bacterial pathogen; well-designed clinical studies have documented the failure of macrolides in treating high-level resistant strains with an MLSB phenotype (*6*). After an 800-mg oral dose, telithromycin achieves serum and epithelial lining fluid concentrations of 2.2 and 15 μg/mL, respectively, yielding a free drug concentration of 0.7 μg/mL in serum and 15 μg/mL in epithelial lining fluid. In an excellent in vitro model, telithromycin eradicated *S. pneumoniae* of the *mefE* phenotype with MICs >0.25 and 1 μg/mL (*7*). The drug was not effective against strains with MICs 2–8 μg/mL, as was seen in our patient. When incubated under CO_2_, MICs of macrolides increase by 1 dilution compared to the MIC in air, against both susceptible and resistant strains. With telithromycin, the MIC increase is 2–6 dilutions but only for macrolide-resistant strains (*8*). The clinical impact of this finding is still to be determined. This report emphasizes the need for routine testing of *S. pneumoniae* isolates for resistance to telithromycin.
